# Effects of foreign direct investment and trade on the prevalence of tobacco consumption in Africa: a panel study

**DOI:** 10.1186/s12992-021-00769-2

**Published:** 2021-10-19

**Authors:** Mustapha Immurana, Micheal Kofi Boachie, Kwame Godsway Kisseih

**Affiliations:** 1 Institute of Health Research, University of Health and Allied Sciences, Ho, Ghana; 2 Department of Health Policy Planning and Management, School of Public Health, University of Health and Allied Sciences, Hohoe, Ghana; 3 Christian Health Association of Ghana Secretariat, Accra, Ghana

**Keywords:** Tobacco consumption, FDI, Trade, Africa

## Abstract

**Background:**

As African governments take measures to enhance international trade and Foreign Direct Investment (FDI) inflows, a major concern is that, these measures can make Africa more vulnerable to the strategies of the tobacco industry. This concern is based on the fact that, each year, tobacco use is estimated to be responsible for the deaths of over eight million people in the world. However, there is very little empirical evidence to refute or confirm the above concern, especially in the African context. This study therefore investigates the effects of FDI and trade on the prevalence of tobacco consumption in Africa.

**Methods:**

Data on a sample of 31 African countries for the period, 2010–2018 are used. The system Generalised Method of Moments (GMM) regression model is employed as the empirical estimation technique.

**Results:**

The findings show that, FDI and trade have negative and positive significant association with the prevalence of tobacco consumption respectively. These findings are robust even after using different specifications and indicators of FDI and trade.

**Conclusion:**

Rising trade (and not FDI) should be of concern to African governments in the quest to reduce the prevalence of tobacco consumption on the continent.

## Background

Over the years, several governments around the world, including those in Africa, have instituted various measures to enhance international trade and Foreign Direct Investment (FDI) inflows. This is because of the economic development benefits that are believed to be associated with trade and FDI inflows. For instance, in 2019, not less than 2654 international investment agreements were in force at the global level. It is therefore not surprising that the global FDI inflows in 2019 amounted to $1.54 trillion [1]. In Africa, similar initiatives have been embarked upon. For instance, Ghana had ratified double taxation agreements with a number of countries, and negotiations on bilateral investment treaties had been completed with 26 countries, as of 2019 [2]. Also, by January 1, 1999, among all developing countries, Egypt had signed the highest number (58) of bilateral investment treaties. At the same time, African countries had completed 335 bilateral investment treaties [3]. It is therefore not farfetched that, recently, FDI inflows as a percentage of Gross Domestic Product (GDP) in Africa increased from 2.13% in 2013 to 2.52% in 2015 [4].

Notwithstanding, one of the major concerns is that, trade and investment agreements that prioritise the right of corporations to free entry and exit in almost all situations, do not pay attention to measures by governments to protect health. Hence, there were situations where nations sent cases to trade tribunals claiming that, public health measures (such as tobacco control) in some countries violated trade rules, and almost all these cases have been successful [5], with the exception of some few cases in for instance, Australia and Uruguay [6, 7]. In addition, trade liberalisation may provide reduced costs benefits to firms, which can lead to lower prices of products including harmful ones such as tobacco. This will therefore lead to a rise in the consumption of tobacco products, which can propel promotional activities of tobacco products by tobacco firms [8].

While increased tobacco consumption may generate profits for corporations, according to the World Health Organization (WHO), tobacco use leads to over eight million deaths annually at the global level. Nonetheless, there are about 1.3 billion tobacco users globally, with 80% of them living in low- and middle-income countries [9]. In Africa, the number of tobacco users has been increasing, from 65 million in 2005 to 71 million in 2015, and this is expected to rise to 80 million by 2025. In addition, relative to other regions, the prevalence of tobacco use in the African region has seen only a marginal decrease. For instance, while the prevalence of tobacco use in the African region decreased by only 5% (18.5 to 13.5%) from 2000 to 2015, that of the Americas and the South-East Asian Regions, fell by 10.7% (30.8 to 20.1%) and 15.4% (46.6 to 31.2%), respectively, in the same period [10]. Thus, the quest for more FDI inflows coupled with the high younger population on the continent, can increase the vulnerability of Africa to the strategies of the tobacco industry [11].

The above therefore calls for an empirical investigation into the effects of trade and FDI on the consumption of tobacco products in Africa. This is particularly important given that, few studies have examined the effects of trade and FDI on tobacco consumption [8, 12–18].

Among these studies, only two of them focused on Africa [8, 16]. Appau, Drope, Labonté et al [8]. examined the relationships between trade liberalisation, tobacco trade and affordability (price) of tobacco, while Immurana, Boachie and Iddrisu [16] examined the effects of tobacco tax and price on smoking prevalence by controlling for FDI. Our study however, investigates the effects of FDI and trade on the prevalence of tobacco consumption in Africa. To our knowledge, this is the first study to analyse the tobacco consumption effects of both trade and FDI inflows in Africa. Doing so, helps in revealing whether trade and FDI have similar or different effects on the percentage of people who use tobacco products in Africa. This will highlight to policy makers the kind of attention to be paid to FDI and trade in designing policies aimed at reducing the prevalence of tobacco consumption in Africa.

## Methods

### Data and variables

In our quest to investigate the effects of FDI and trade on the prevalence of tobacco consumption in Africa, we source data from the World Bank’s World Development Indicators (WB’s WDI) as well as the WHO. The data cover 31 African countries (see Appendix) for the period 2010 to 2018. The study period and the number of countries are largely dictated by data availability on variables, especially the prevalence of tobacco consumption. Linear interpolation is employed to fill gaps in the data.

In this study, the dependent variable is the prevalence of current tobacco consumption or use. The main independent variables are FDI and trade (trade openness and imports). Also, tobacco taxes, income and education are used as control variables. Details of how these variables are measured and their expected signs are shown in Table 1.

**Table 1:** Measurements and expected signs of variables

Variable	Definition/measurement	Source	Expected sign
Prevalence of tobacco consumption	The percentage of people aged 15 years and above who currently use any tobacco product	WB’s WDI	Not applicable
FDI	Net inflows of FDI as a percentage of GDP	WB’s WDI	+/−
Trade	1. Imports plus exports as a share of GDP (Trade openness) 2. Imports as a share of GDP	WB’s WDI	+/−
Tobacco tax	Total tax on a pack of 20 cigarettes expressed as a percentage of the retail cost (price) of the most popular (mostly sold) brand of cigarettes. This indicator is used because we do not have data on tobacco taxes. In addition, using cigarette tax to proxy tobacco tax is justified because according to the WHO [19], cigarettes are the most widely used tobacco products	WHO	–
Education	Gross primary school enrolment as a ratio of the total population of individuals in the official age category for primary education	WB’s WDI	–
Income	Growth rate of per capita GDP	WB’s WDI	+/−

‘+’ means will increase the prevalence of tobacco consumption; ‘–’ means will decrease the prevalence of tobacco consumption

With regard to the expected signs of the variables (see Table 1), the effects of FDI and trade on the prevalence of tobacco consumption are uncertain or mixed. Thus, if FDI inflows are geared towards the tobacco industry, it may increase the production and hence, consumption of tobacco products [15]. This is because, when FDI inflows into the tobacco sector increase the production/supply of tobacco, given that demand for tobacco products remains unchanged, tobacco firms would end up reducing the prices of their products in order to clear the surplus. This fall in price will lead to a rise in the consumption of tobacco products, all other things being equal. In addition, investment agreements may limit governments’ ability to strengthen measures towards tobacco control for fear of cost of litigation, since some investors may challenge these measures in trade tribunals [5, 8]. Moreover, FDI in the tobacco sector can provide foreign firms with vibrant local presence which makes them more capable of lobbying government officials in their favour [18].

Concerning trade liberalisation, since it can provide some cost reduction benefits to tobacco firms along the supply chain, these firms can transfer the benefits into lower prices of their products, which will increase the consumption of tobacco products, all other things being equal [8]. Moreover, trade liberalisation can enhance competition among tobacco firms, and in the quest by these firms to increase demand for their products, they may end up reducing the prices of their products as well as increasing advertisement and branding. These, have the potential to increase the consumption of tobacco products, all other things being equal [14]. For instance, recently in Africa, there is evidence that, in push carts and convenience stores, tobacco firms are using marketing/advertising strategies such as flavoured cigarettes, single cigarettes and smaller packs to lure school going children to smoke [20]. Conversely, if the already existing (as well as future) inflows of FDI and trading activities are diverted from the tobacco sector to other sectors, the expectation is that, the production and consumption of tobacco products will fall, all other things being equal.

Tobacco tax is expected to decrease the consumption of tobacco products via a rise in price [16, 21–23]. Income is expected to have a mixed effect on tobacco consumption. Thus, as income increases, it will decrease and increase the consumption of tobacco products if tobacco products are inferior and normal goods respectively [16]. As regards education, we expect it to decrease the consumption of tobacco products. Thus, education can make people to be more willing to utilise and disregard health enhancing as well as health deteriorating products (such as of tobacco) respectively [see [24–29]].

**Fig. 1:**
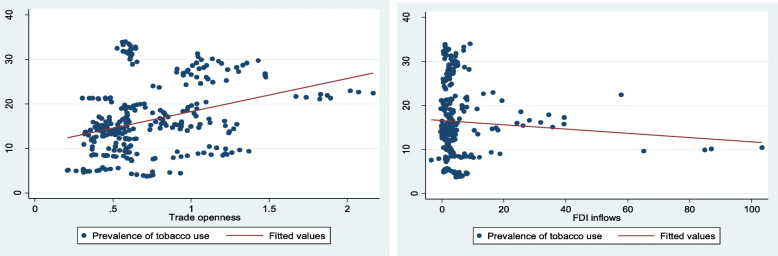
The relationships between trade, FDI and tobacco consumption in 31 African countries (2010–2018)

A graphical presentation (Fig. 1) of the data shows that, whiles there exist a positive relationship between trade and the prevalence of tobacco consumption among the selected countries, the relationship between FDI and the prevalence of tobacco consumption is negative. This therefore reinforces the need for a multivariate analysis of the effects of trade and FDI on the prevalence of tobacco consumption in the selected countries.

### Statistical analysis

To examine the effects of FDI and trade on the prevalence of tobacco consumption, we specify a simple equation as follows:

TB = *f*(FDI, Trade, X) (1),

where the prevalence of tobacco consumption (TB) is expressed as a function of FDI, Trade and a vector of control variables (X). We therefore re-specify eq. 1 in an estimable form as follows:

TB_it_ = ϖ + ƛTB_it − 1_ + *α*FDI_it − 1_+ ΨTrade_it − 1_ + ϢX_it_ + ȹ_t_ + ε_it_ (2),

where ϖ is the intercept of the equation, and *α*, Ψ as well as Ϣ are coefficients of their respective variables. Also, i, t, ȹ and ε represent the individual countries, time (year), time fixed effects (year dummies) and the white noise disturbance term, respectively. We use the first lags of FDI (FDI_it − 1_) and Trade (Trade_it − 1_) because it will take time for them to affect the current prevalence of tobacco consumption. The time fixed effects are also used in order to deal with cross-sectional dependence as well as unanticipated situations or occurrences that may affect the prevalence of tobacco consumption [30, 31]. Also, we introduce the first lag of TB (TB_it − 1_) to capture the persistence of prevalence of tobacco consumption overtime. This is because, the previous levels of tobacco consumption may influence current tobacco consumption. Thus, ƛ becomes the coefficient of the first lag of tobacco consumption.

Since we have two measures of trade (imports plus exports as a share of GDP (Trade openness) and imports as a share of GDP), we run eq. 2 for each trade measure.

Given the nature of eq. 2, the persistence or dynamic term introduced may be correlated with the white noise disturbance term, which can lead to endogeneity, making results from estimators such as the Ordinary Least Square (OLS), Fixed Effects (FE) and Random Effects (RE) inconsistent and biased [32–34]. Moreover, there is the likelihood of the prevalence of tobacco consumption influencing some of the right-hand side variables leading to endogeneity. For instance, rising prevalence of tobacco consumption may direct trading and investment activities towards the tobacco industry. Also, rising prevalence of tobacco consumption may force governments to impose or increase tobacco taxes. Ignoring these endogeneity concerns may lead to biased and unreliable estimates.

Since getting external instruments for these potentially endogenous variables is very difficult, it makes it less feasible to use Instrumental Variable (IV) estimators such as the Two-Stage Least Square (2SLS), IVFE and IVRE. Moreover, these estimators are not dynamic in nature.

This study therefore uses the dynamic panel system Generalised Method of Moments (GMM) of Arellano and Bover [35] and Blundell and Bond [36] as the estimation technique (albeit its restrictive assumptions and limitations) [32, 35–37] . The system GMM uses both level and first-differenced equations, and hence employs lagged levels and first differences of variables as instruments via additional moment conditions [38]. Thus, the system GMM makes it possible to use internal variables as instruments in order to deal with potential endogeneity issues.

However, in order for the estimates to be appropriate, the instruments must be valid and the first-differenced errors should not exhibit second-order serial correlation. The Hansen *J* test of overidentification or over-identifying restrictions (Hansen) and the Arellano-Bond serial correlation test (AR (2)) are therefore used to confirm the validity of the instruments as well as the estimates. The insignificance of the *p*-values of these tests therefore confirms the validity of the instruments and the estimates [30, 32, 39–41]. Moreover, as suggested by Roodman [32], we ensure that the number of instruments is less than the number of cross-sections (countries) in order to avoid the proliferation of instruments which may weaken the ability of the Hansen *J* overidentification test to detect the invalidity of the instruments, as well as overfit variables that are endogenous, hence failing to remove their endogenous aspects.

To check the robustness of our results, we first run eq. 2 with only trade openness and the control variables, second, with only imports and the control variables, and third, with only FDI inflows and the control variables. Last but not the least, we run a final model by employing the KOF trade globalisation index [42, 43] and the Chinn-Ito index of capital openness [44] as proxies for trade and FDI respectively.

## Results

In this section, we present the regression results (two-step system GMM estimates) of the effects of FDI and trade on the prevalence of tobacco consumption among the 31 selected African countries. The Appendix presents summary statistics of variables used, correlation matrix and average trade, FDI and prevalence of tobacco use per country. As evident in Tables 2 and 3, in all our models, the Hansen *J* and the AR (2) tests results as well as the absence of the proliferation of instruments confirm the appropriateness of our instruments and estimates. Moreover, all our models have high goodness of fit.

**Table 2:** Effects of FDI and trade on the prevalence of tobacco consumption (Baseline results)

	(1)	(2)
	Prevalence of tobacco use	Prevalence of tobacco use
L.Prevalence of tobacco use	0.966^***^	0.973^***^
	(0.00966)	(0.0129)
Tobacco tax	0.00310	0.00399
	(0.00392)	(0.00504)
L.Trade openness	0.572^**^	
	(0.242)	
L.FDI inflows	−0.0168^**^	−0.0135^*^
	(0.00650)	(0.00677)
Income	0.00102	0.00109
	(0.0168)	(0.0135)
Education	0.00173	0.00221
	(0.00319)	(0.00322)
L.Imports		0.00405^*^
		(0.00238)
Constant	−0.378	−0.368
	(0.406)	(0.377)
Observations	216	216
Countries	31	31
Instruments	22	22
AR(2)	0.937	0.425
AR(2) *p*-value	0.349	0.671
Hansen	4.188	6.919
Hansen *p*-value	0.899	0.646
F stat	6871.8	8170.2
F stat *p*-value	0.000	0.000

Standard errors in parentheses; ^*^ *p* < 0.1, ^**^ *p* < 0.05, ^***^ *p* < 0.01; L. refers to the first lag of the respective variable; Year dummies are not reported for brevity. The null hypotheses of the Hansen and the AR (2) tests state the joint validity of the instruments and the absence of second-order serial correlation respectively [30, 32]

**Table 3:** Effects of FDI and trade on the prevalence of tobacco consumption (Robustness checks)

	(1)	(2)	(3)	(4)
	Prevalence of tobacco use	Prevalence of tobacco use	Prevalence of tobacco use	Prevalence of tobacco use
L.Prevalence of tobacco use	0.975^***^	0.963^***^	0.962^***^	0.994^***^
	(0.0234)	(0.00973)	(0.0195)	(0.0197)
Tobacco tax	0.00520	−0.00604^*^	− 0.00135	− 0.00909
	(0.00561)	(0.00349)	(0.0125)	(0.00845)
L.Trade openness	0.167^*^			
	(0.0845)			
Income	0.00760	0.0205^*^	0.0425^*^	−0.00524
	(0.00698)	(0.0119)	(0.0212)	(0.0105)
Education	0.0138^***^	0.0113^***^	0.00298	−0.0112
	(0.00299)	(0.00260)	(0.00826)	(0.00771)
L.Imports		0.00733^**^		
		(0.00348)		
L.FDI inflows			−0.00727^*^	
			(0.00393)	
L.KOF trade globalisation index				0.0115^**^
				(0.00442)
L.Chinn-Ito index of capital openness				−0.0741^*^
				(0.0420)
Constant	−1.688^***^	−1.046^***^	−0.0109	0.817
	(0.204)	(0.247)	(0.723)	(0.720)
Observations	216	216	217	217
Countries	31	31	31	31
Instruments	24	27	21	24
AR(2)	−1.233	0.372	−1.097	−1.379
AR(2) *p*-value	0.218	0.710	0.273	0.168
Hansen	13.32	11.57	4.151	8.980
Hansen *p*-value	0.346	0.712	0.901	0.774
F stat	15,428.4	7834.9	2903.3	5706.0
F stat *p*-value	0.000	0.000	0.000	0.000

Standard errors in parentheses; ^*^ *p* < 0.1, ^**^ *p* < 0.05, ^***^ *p* < 0.01; L. refers to the first lag of the respective variable; Year dummies are not reported for brevity; The null hypotheses of the Hansen and the AR (2) tests state the joint validity of the instruments and the absence of second-order serial correlation respectively [30, 32]

In Table 2 (baseline results), we find that, the past year's prevalence of tobacco consumption has a positive significant association (at the 1% level) with the current prevalence of tobacco consumption. Thus, a rise in the previous year’s level of tobacco consumption is found to be significantly associated with an increase in the current level of tobacco consumption.

Turning to the main variables of interest, we find that, the past year’s level of trade openness has a positive significant relationship with the current prevalence of tobacco consumption, while that of FDI is found to be negatively significant (Table 2, model 1). Specifically, a unit increase in the past year’s level of trade openness is associated with an increase in the current prevalence of tobacco consumption by 0.57 units at the 5% level of significance. Nonetheless, a unit increase in the previous year’s FDI inflows is associated with a 0.02 units fall in the current prevalence of tobacco consumption at the 5% level of significance. Moreover, in model 2 (Table 2), while the past year’s level of FDI still maintains the same negative significant relationship (− 0.01 at 10% level of significance) with the prevalence of tobacco consumption, the past year’s level of imports is found to be associated with an increase in the current prevalence of tobacco consumption by 0.004 units at the 10% level of significance.

In Table 3 (robustness checks), the past year’s level of the prevalence of tobacco consumption is found to be associated with an increase in the current prevalence of tobacco consumption at the 1% level of significance in all models.

As regards the main variables of interest, running them in separate models, we find that, their signs and significance are qualitatively the same as those in the baseline results. Specifically, the past year’s levels of trade openness and imports are found to be associated with an increase in the current prevalence of tobacco consumption by 0.17 units (Table 3 model 1) and 0.007 units (Table 3 model 2) at the 10 and 5% level of significance respectively. Conversely, the past year’s level of FDI inflows is found to have a negative significant relationship (− 0.007 at the 10% level of significance) with the current prevalence of tobacco consumption.

For further robustness checks, we use the KOF trade globalisation index and the Chinn-Ito index of capital openness as proxies for trade and FDI respectively, and the findings are not qualitatively different from the results of the other indicators. Specifically, while the past year’s value of the KOF trade globalisation index is found to be associated with an increase in the current prevalence of tobacco consumption by 0.01 units at the 5% level of significance, the past year’s level of the Chinn-Ito index of capital openness is found to be associated with a decrease in the current prevalence of tobacco consumption by 0.07 units at the 10% level of significance.

## Discussion

In this study, we investigate the effects of FDI and trade on the prevalence of tobacco consumption in 31 African countries. We find that, the lagged prevalence of tobacco consumption variable has a positive significant relationship with the current prevalence of tobacco consumption.

Concerning the main variables of interest, while trade openness and imports (Trade) are found to be associated with an increase in the prevalence of tobacco consumption, the relationship between FDI and the prevalence of tobacco consumption is negative. These findings are robust even after running separate models for FDI, trade openness and imports as well as proxying trade and FDI by the KOF trade globalisation index and the Chinn-Ito index of capital openness respectively. It is therefore not surprising that, in our sample, countries like Botswana, Lesotho, Mauritius, Seychelles, South Africa and Tunisia that have relatively high levels of trade liberalisation, have relatively high prevalence of tobacco use, while countries such as Congo Republic, Ghana, Liberia and Nigeria with relatively high net FDI inflows, are found to have relatively low prevalence of tobacco use (see Appendix). The finding on trade could be that, trade liberalisation makes tobacco products readily and cheaply available in African countries, which can increase the prevalence of tobacco use [8]. On the other hand, the finding on FDI inflows reducing the prevalence of tobacco use could be that, FDI inflows into the African continent are directed towards other sectors instead of the tobacco industry. For instance, data shows that, recent FDI inflows into Africa are more concentrated in the services sector rather than the manufacturing sector [45]. The result on trade is similar to those of Chaloupka and Laixuthai [12] who found that, the opening up of markets in Japan, Thailand, South Korea and Taiwan to United States (US) cigarettes, increased the per capita consumptions of cigarettes in these countries by nearly 10%. Similarly, Honjo and Kawachi [13] found trade liberalisation to increase smoking in Japan, while Taylor, Chaloupka, Guindon et al [14] found trade liberalisation to increase cigarette smoking in low-and middle-income countries. However, in Southeast Asia, there was no clear link between trade liberalisation and tobacco consumption [17]. The outcome on FDI conflicts findings from the former Soviet Union [15]. Nonetheless, the finding is similar to a previous study on Africa which found the effect of FDI on smoking prevalence to be negative but insignificant for a sample of 24 countries [16]. The differences in significance could be due to the use of relatively more countries in the present study. The finding suggests that, FDI inflows into Africa, do not promote tobacco consumption, and this could improve public health. This corroborates earlier results by Immurana [46] who found FDI to improve population health in Africa.

In spite of the above, the present study is limited to only 31 countries and hence caution should be exercised in generalising the findings to be representative of the entire African continent.

## Conclusion

As African governments initiate a number of measures towards enhancing international trade and FDI inflows, a major concern is that, these measures can make Africa more vulnerable to the strategies of the tobacco industry. Meanwhile, each year, tobacco use is responsible for the deaths of more than eight million people in the world. In this study, we therefore provide the foremost empirical evidence of the effects of FDI and trade on the prevalence of tobacco consumption in the African context using data from 2010 to 2018 on 31 countries. Our findings from two-step system GMM estimations show that, while FDI is associated with a reduction in tobacco consumption, trade on the other hand is associated with an increase in the consumption of tobacco products. These findings are robust even after using both different specifications and indicators of trade and FDI. It is therefore not surprising that in our sample, countries with high trade liberalisation and net FDI inflows have relatively high and low prevalence of tobacco use respectively. The policy implication is that, as governments in Africa institute measures towards enhancing trade, conscious efforts must be made to ensure that trade does not increase the prevalence of tobacco consumption on the continent.

## Data Availability

The datasets supporting the conclusions of this article are available from the websites of the World Health Organization (https://www.who.int/tobacco/global_report/2017/appendix-ix/en/; https://www.who.int/tobacco/global_report/en/) and the World Bank (https://databank.worldbank.org/source/world-development-indicators#advancedDownloadOptions).

## Appendix

**Table 4:** List of countries

Algeria	Ethiopia	Mauritius	Seychelles
Benin	Gambia, The	Morocco	South Africa
Botswana	Ghana	Mozambique	Tanzania
Burkina Faso	Kenya	Namibia	Togo
Burundi	Lesotho	Niger	Tunisia
Congo, Rep.	Liberia	Nigeria	Uganda
Cote d’Ivoire	Madagascar	Rwanda	Zimbabwe
Egypt, Arab Rep.	Mali	Senegal	

**Table 5:** Descriptive Statistics

Variable	Obs	Mean	Std. Dev.	Min	Max
Prevalence of tobacco use	279	16.32	7.698	3.7	34
Tobacco tax	279	42.62	19.27	4.88	85.96
Trade openness	277	.742	.361	.207	2.165
Imports	277	43.917	22.352	10.666	117.154
FDI inflows	279	5.636	11.918	−3.374	103.337
Income	279	2.203	3.347	−12.442	18.066
Education	242	106.754	18.324	62.708	148.23
KOF trade globalisation index	279	46.737	14.015	24.356	84.207
Chinn-Ito index of capital openness	279	−.39	1.394	−1.92	2.334

**Table 6:** Matrix of correlations

Variables	(1)	(2)	(3)	(4)	(5)	(6)	(7)	(8)	(9)
(1) Prevalence of tobacco consumption	1.000								
(2) Tobacco tax	0.742	1.000							
(3) Trade openness	0.322	0.242	1.000						
(4) Imports	0.246	0.154	0.943	1.000					
(5) FDI inflows	−0.071	−0.125	0.385	0.484	1.000				
(6) Income	−0.139	−0.139	0.155	0.121	0.131	1.000			
(7) Education	0.266	0.205	0.093	0.111	−0.017	−0.016	1.000		
(8) KOF trade globalisation index	0.597	0.576	0.686	0.559	0.128	0.097	0.222	1.000	
(9) Chinn-Ito index of capital openness	0.105	0.271	0.272	0.271	0.229	0.017	−0.076	0.158	1.000

**Table 7:** Average trade, FDI and prevalence of tobacco use per country (2010–2018)

Country	Trade openness	Imports	FDI	Prevalence of tobacco consumption
Algeria	0.62	31.93	0.8	19.42
Benin	0.57	31.03	1.77	8.71
Botswana	1	50.9	1.56	24.97
Burkina Faso	0.59	32.59	1.9	18.07
Burundi	0.39	30.92	1.07	14.02
Congo, Rep.	1.14	56.22	16.38	14.73
Cote d’Ivoire	0.7	32.63	1.29	14.21
Egypt, Arab Rep.	0.41	24.65	1.96	21.26
Ethiopia	0.39	28.12	3.14	5.02
Gambia, The	0.5	31.58	1.87	15.69
Ghana	0.75	41.8	6.5	4.16
Kenya	0.48	30.25	1.71	13.06
Lesotho	1.37	94.49	4.11	27.77
Liberia	1.23	97.91	42.74	9.36
Madagascar	0.59	33.36	5.29	31.31
Mali	0.6	36.33	2.81	12.14
Mauritius	1.07	59.3	3.36	28.37
Morocco	0.82	46.69	2.64	16.34
Mozambique	0.99	66.7	25	16.69
Namibia	0.97	57.71	4.68	19.01
Niger	0.44	29.57	6.76	8.36
Nigeria	0.34	14.51	1.17	5.22
Rwanda	0.45	30.58	3.11	14.56
Senegal	0.58	35.89	2.23	10.07
Seychelles	1.9	100.66	15.36	21.96
South Africa	0.61	30.43	1.15	32.57
Tanzania	0.45	26.41	3.29	15.07
Togo	0.94	55.54	3.85	8.36
Tunisia	1.01	55.6	2.24	28.57
Uganda	0.38	23.68	3	12.17
Zimbabwe	0.63	39.76	1.97	14.74
